# Foslevodopa/Foscarbidopa in advanced Parkinson's disease: One-year continuation

**DOI:** 10.1016/j.prdoa.2026.100530

**Published:** 2026-11-06

**Authors:** Naoki Takao, Makoto Shiraishi, Hidetaka Naganuma, Takayuki Kawazawa, Sakae Hino, Yoshihisa Yamano

**Affiliations:** Department of Neurology, St. Marianna University School of Medicine, Kawasaki, Japan

**Keywords:** Parkinson's disease, Foslevodopa/foscarbidopa, Device-aided therapy, Treatment persistence, Adverse events

## Abstract

**Objective:**

Continuous subcutaneous foslevodopa/foscarbidopa (LDp/CDp) infusion is a non-surgical device-aided therapy for advanced Parkinson's disease (PD). We retrospectively analyzed 1-year treatment persistence, changes in oral medication burden, and safety.

**Methods:**

Twenty-one patients with advanced PD in whom LDp/CDp infusion was initiated between October 2023 and October 2025 were evaluated for levodopa equivalent daily dose (LEDD), oral levodopa-containing compound (LCC) dose, infusion rate settings, and psychiatric and skin-related adverse events. Baseline characteristics were compared between the 1-year continuation group (*n* = 16) and the discontinuation group (*n* = 5).

**Results:**

In the selected single-center cohort, 16 of the 21 patients continued this therapy at the 12-month mark. Among patients for whom long-term assessment was possible, a reduction in the oral LCC dose was observed. All five discontinuations were directly attributable to psychiatric symptoms that emerged or worsened within 1 month of initiation; concomitant skin-related events were observed but were not decisive. Total LEDD increased from 1169.0 to 1354.8 mg/day at 12 months. Psychiatric symptoms were frequently recorded early in the treatment course, whereas skin indurations were frequently observed at assessment time points from the mid-treatment period onward.

**Conclusion:**

LDp/CDp showed treatment persistence and sustained reduction in oral LCC burden over one year. Management should be adapted to the treatment phase: psychiatric symptoms cluster early, whereas skin-related events accumulate over the mid-to-long term.

## 1 Introduction

When treating Parkinson's disease (PD), optimizing dopamine replacement therapy becomes extremely difficult as the disease progresses [1], [2]. After the early “honeymoon phase,” motor complications such as wearing-off and dyskinesia emerge as the brain's ability to store dopamine declines and the blood concentration of oral levodopa fluctuates [3], significantly impairing patients' activities of daily living and quality of life (QOL) [4]. For advanced PD, frequent administration of oral medications and combination therapy are attempted, but delayed gastric emptying and unstable gastrointestinal absorption can limit the achievement of adequate symptom control [5], [6].

To address these challenges, device-aided therapies (DATs) such as deep brain stimulation (DBS) and levodopa-carbidopa intestinal gel (LCIG) have been introduced, but the surgical invasiveness associated with these procedures remains a barrier to their adoption [7], [8]. Approved in Japan in 2022 and launched in July 2023, continuous subcutaneous infusion therapy of foslevodopa/foscarbidopa (LDp/CDp) has enabled the maintenance of stable levodopa blood levels through 24-h continuous delivery as a non-invasive “third DAT” that does not require surgery. Clinical trials have reported prolonged on time, reduced off time, and improved sleep and QOL, demonstrating excellent efficacy [9], [10].

However, practical implementation of LDp/CDp continuous subcutaneous infusion therapy in clinical practice has highlighted challenges not fully apparent in the clinical trials. In particular, skin-related adverse events at the infusion site, such as induration, erythema, and infection, and psychiatric symptoms associated with changes in dopamine stimulation, such as hallucinations and delusions, are important factors influencing treatment continuation and raise concerns about early treatment discontinuation [11]. In Japan, a multicenter real-world clinical study involving 108 patients across seven institutions reported on treatment continuation and early discontinuation of LDp/CDp therapy [12]. However, the temporal patterns of psychiatric and skin-related adverse events and specific implementation protocols remain to be fully elucidated.

At our hospital's Parkinson's Disease Treatment Center, upon initiating LDp/CDp continuous subcutaneous infusion therapy, we established a protocol based on multidisciplinary collaboration and conducted detailed monitoring on a patient-by-patient basis. The present study was conducted to retrospectively analyze the 12-month course of 21 patients treated at our hospital to clarify 1) treatment persistence, 2) changes in oral levodopa/carbidopa combination (LCC) dosage and infusion rate, 3) temporal occurrence of psychiatric symptoms and skin-related adverse events, and 4) actual implementation of the protocol, which includes a lower initial infusion rate, multidisciplinary collaboration, symptom diaries, and photographic records of infusion sites. Through this analysis, we examined the clinical requirements contributing to the long-term, stable continuation of LDp/CDp continuous subcutaneous infusion therapy and present practical insights into the implementation of DAT in the treatment of advanced PD. The purpose of this study was to retrospectively describe, based on our institution's experience with the introduction of this therapy, the distribution over time of treatment continuation status, oral LCC dosage, flow rate settings, and adverse events recorded in the medical records.

## 2 Methods

### 2.1 Patients

This study included a consecutive series of 21 patients with advanced-stage PD who began long-acting subcutaneous LDp/CDp infusion therapy at our hospital between October 2023 and October 2025, incorporating all cases initiated during the study period. For patients whose daily lives were impaired by motor complications despite adjustments to oral medication, a multidisciplinary team evaluated the feasibility of initiating this therapy. The therapy was selected based on available treatment options—including DBS and LCIG—the indications for each treatment, and the patient's wishes. Patients with severe cognitive impairment or active psychotic symptoms, as well as those who were unable to manage the infusion device independently and could not receive support from caregivers or others, were excluded from consideration. Cognitive function was not assessed solely based on scale scores; instead, it was evaluated comprehensively, taking into account the patient's ability to operate the device and the available support environment. The patients' background characteristics (median [interquartile range: IQR] (minimum–maximum)) included age 69.0 [60.0–75.0] (53.0–86.0) years, disease duration of 13.0 [10.0–17.0] (5.0–37.0) years, and Hoehn-Yahr stage of 3.0 [2.0–3.0] (1.0–4.0) in the on state. The Mini-Mental State Examination (MMSE) score assessing cognitive function prior to treatment initiation was 29.0 [27.0–30.0] (20.0–30.0) points, indicating that overall cognitive function of the patients was generally preserved. Patient demographics and concomitant medications prior to the start of treatment (defined as the medications prescribed before any discontinuation or adjustment upon hospital admission) are summarized in Table 1.

**Table 1:** Baseline characteristics of the 21 study patients overall and comparison between the continuation and discontinuation groups.

Variable	Total (*n* = 21)	Continuation group (*n* = 16)	Discontinuation group (*n* = 5)	*P* value
	Median [IQR] (min–max)	Median [IQR] (min–max)	Median [IQR] (min–max)	
Age, years	69.0 [60.0–75.0] (53.0–86.0)	68.0 [58.0–75.2] (53.0–86.0)	69.0 [68.0–71.0] (60.0–78.0)	0.741
Sex, male/female, n (%)	14 (66.7%) / 7 (33.3%)	11 (68.8%) / 5 (31.2%)	3 (60.0%) / 2 (40.0%)	1.00
Disease duration, years	13.0 [10.0–17.0] (5.0–37.0)	11.5 [8.8–15.5] (5.0–37.0)	14.0 [14.0–18.0] (13.0–20.0)	0.126
Hoehn-Yahr stage (on state)	3.0 [2.0–3.0] (1.0–4.0)	2.5 [2.0–3.0] (1.0–3.0)	3.0 [2.0–3.0] (2.0–4.0)	0.391
MMSE score, points	29.0 [27.0–30.0] (20.0–30.0)	29.0 [27.0–30.0] (20.0–30.0)	27.0 [27.0–28.5] (27.0–30.0)	0.823
Oral LCC before initiation, mg/day	600.0 [400.0–800.0] (300.0–1270.0)	525.0 [350.0–800.0] (300.0–1270.0)	940.0 [700.0–1200.0] (600.0–1200.0)	0.062
Total LEDD before initiation, mg/day	1169.0 [900.0–1475.0] (800.0–2374.2)	1167.0 [873.5–1448.8] (800.0–2374.2)	1199.2 [1066.0–1512.0] (900.0–1659.1)	0.548

**Concomitant medications before initiation, n (%**)				
**Dopamine agonists**	17 (81.0%)	14 (87.5%)	3 (60.0%)	0.228
Pramipexole	4 (19.0%)	3 (18.8%)	1 (20.0%)	1.000
Rotigotine	8 (38.1%)	6 (37.5%)	2 (40.0%)	1.000
Ropinirole	5 (23.8%)	5 (31.3%)	0 (0.0%)	0.280
**COMT inhibitors**	16 (76.2%)	13 (81.3%)	3 (60.0%)	0.292
Entacapone	5 (23.8%)	2 (12.5%)	3 (60.0%)	0.057
Opicapone	11 (52.4%)	11 (68.8%)	0 (0.0%)	0.031
**MAO-B inhibitors**	12 (57.1%)	10 (62.5%)	2 (40.0%)	0.601
Selegiline	2 (9.5%)	2 (12.5%)	0 (0.0%)	1.000
Rasagiline	7 (33.3%)	5 (31.3%)	2 (40.0%)	1.000
Safinamide	3 (14.3%)	3 (18.8%)	0 (0.0%)	0.548
**Zonisamide**	8 (38.1%)	5 (31.3%)	3 (60.0%)	0.311
**Istradefylline**	7 (33.3%)	6 (37.5%)	1 (20.0%)	1.000
**Psychotropic drugs**	0 (0.0%)	0 (0.0%)	0 (0.0%)	1.000

Abbreviations: COMT, catechol-*O*-methyltransferase; IQR, interquartile range; LCC, levodopa-containing compound; LEDD, levodopa equivalent daily dose; MAO-B, monoamine oxidase B; MMSE, Mini-Mental State Examination.

All values are shown as median [interquartile range] (minimum–maximum) unless otherwise indicated. The continuation group comprised patients who maintained treatment for 1 year, and the discontinuation group comprised those who dropped out because of early adverse events. Between-group comparisons were performed using the Mann-Whitney *U* test for continuous variables and Fisher's exact test for categorical variables.

### 2.2 Data collection and evaluation items

Clinical information was retrospectively extracted from the patients' electronic medical records. Data collected included temporal trends in the levodopa equivalent daily dose (LEDD), the oral LCC dosage, and the infusion rate program settings for the LDp/CDp continuous subcutaneous infusion therapy (standard setting, low setting) from before initiation through 12 months after initiation (immediately after initiation and at 1, 3, 6, and 12 months thereafter). To evaluate safety, adverse events were investigated in detail. The types of events and the number of patients with each event at each time point were recorded for skin-related adverse events (pain, erythema, induration, cellulitis/suppuration). Adverse events were retrospectively identified from the available medical records, including physicians' medical records, nursing records, symptom logs, and photographic records of puncture sites. Psychiatric symptoms were classified into the following categories: hallucinations (including visual hallucinations); agitation and excitement; depression; dopamine dysregulation syndrome; and delusions. The severity of skin-related adverse events was retrospectively assessed in accordance with CTCAE v5.0, based on medical record entries and photographs of the puncture sites. To evaluate the impact of these adverse events on treatment continuation, the occurrence of treatment discontinuation (withdrawal) and its contributing factors were also analyzed.

### 2.3 Initiation protocol and dose adjustment

When initiating LDp/CDp continuous subcutaneous infusion therapy, we used a standardized protocol developed through multidisciplinary collaboration at our institution. Starting before hospitalization, physicians and nurses collaborated to share information and repeatedly provided advance explanations to patients and caregivers regarding the risks of adverse events and the time lag—typically 2 to 3 months—required for stable therapeutic effects to manifest. To facilitate learning of the procedure, nurses performed the initial administration alongside caregivers, but from the second administration onward, we encouraged patients to learn the procedure independently by using instructional videos produced in-house. We also established an objective monitoring system by instructing patients to keep symptom diaries and by photographing the skin condition at the infusion site for inclusion in the medical records.

Regarding dosage settings, we generally used the “standard setting”—calculated based on pre-initiation LEDD—as a baseline. However, to prioritize safety during the initial implementation phase, we made appropriate adjustments to start with a “low setting” equivalent to 70–80% of the calculated value. Of note, no loading doses were administered to any of the patients in this study. In one patient, the infusion was suspended overnight at the patient's request rather than continued at a reduced nocturnal rate. As a general rule, concomitant medications such as catechol-*O*-methyltransferase (COMT) inhibitors and monoamine oxidase B (MAO-B) inhibitors were uniformly discontinued on the day before the start of infusion (the day of admission), in accordance with our institution's protocol. At the same time, certain oral LCCs and dopamine agonists were replaced with LDp/CDp continuous subcutaneous infusion therapy.

Upon discharge, the inpatient nurse provided a detailed handover to the outpatient nurse regarding the patient's mastery of self-administration techniques and any concerns. After discharge, outpatient care that included clinical assessments of motor and psychiatric symptoms, fine-tuning of infusion rates, and psychological support was continued. However, standardized assessments using scales such as the MDS-UPDRS and PDQ-39 were not systematically collected from the entire study population at a specific point in time and were therefore not included in the outcomes of this study.

### 2.4 Statistical analysis

Given the limited number of patients, continuous variables are presented as medians and IQR, and nonparametric tests were used for statistical analysis. The Friedman test was used to compare changes over time in LEDD and oral LCC dosage, and a Wilcoxon signed-rank test with Bonferroni correction was performed as a post hoc analysis. Missing values were not imputed. Descriptive statistics for each time point were calculated based on patients for whom data were available at that time (available-case analysis: immediately after initiation and at 1 month, *n* = 21; at 3 months, *n* = 16; at 6 and 12 months, *n* = 15). One patient who was transferred to another hospital between the 3- and 6-month evaluations was included in the descriptive statistics up to the 3-month time point but excluded from those for subsequent time points. The Friedman test and post hoc analyses were performed on the 15 patients for whom data were available for all six time points.

To evaluate in an exploratory manner background factors potentially associated with treatment continuation, a comparison was performed between the “continuation group” (*n* = 16), in which treatment was maintained for one year, and the “discontinuation group” (*n* = 5), in which patients dropped out due to early adverse events. The Mann-Whitney *U* test was used to compare continuous variables between the two groups, and Fisher's exact test was used to compare categorical variables. The pre-initiation baseline data for these two groups are summarized in Table 1. Statistical significance was defined as *P* < 0.05 in two-sided tests. EZR was used for the analysis [13].

## 3 Results

### 3.1 Patient characteristics and treatment continuation rate

This study included 21 patients with advanced PD in whom continuous subcutaneous LDp/CDp infusion was initiated at our hospital. The baseline data for all patients are shown in Table 1. The median age of the patients (14 men [66.7%], 7 women [33.3%]) was 69.0 [IQR: 60.0–75.0] (range: 53.0–86.0) years, and their median disease duration was 13.0 [10.0–17.0] (5.0–37.0) years. The median oral LCC dose prior to initiation was 600.0 [400.0–800.0] (300.0–1270.0) mg/day, and the median pre-initiation LEDD was 1169.0 [900.0–1475.0] (800.0–2374.2) mg/day.

During the 1-year follow-up period after treatment initiation, 16 of the 21 patients (76.2%) continued treatment, and 5 patients (23.8%) discontinued treatment (dropouts). In these 5 patients, the direct primary cause of discontinuation was psychiatric symptoms (such as depression, hallucinations, or agitation) that emerged or worsened during the initial phase (within 1 month) of treatment. Although mild-to-moderate skin-related events at the infusion site (mild erythema or pain) were also observed concurrently, in none of these patients did the skin-related events themselves constitute a direct reason for discontinuation leading to withdrawal from treatment.

As all 5 of these patients discontinued treatment after the completion of clinical evaluations and data collection at the 1-month mark, the analyses conducted immediately after initiation and at 1 month included all 21 patients; however, all 5 patients had withdrawn by the 3-month mark, resulting in 16 patients in the continuation group at 3 months. Of the 16 patients in the continuation group, 4 (25.0%) were transferred to other hospitals due to relocation to distant areas, with all continuing this therapy at their new hospitals. Of these, 3 were transferred after completing the 12-month evaluation, and there were no missing data. The remaining patient transferred between the 3- and 6-month evaluations, so data for this patient at the 6- and 12-month time points were not available from our hospital. Therefore, the analysis at the 6- and 12-month time points included 15 patients.

The pre-initiation clinical backgrounds of the continuation group (*n* = 16) and the discontinuation group (*n* = 5) can be compared in Table 1. The results showed that the discontinuation group tended to receive higher pre-initiation oral LCC doses compared to the continuation group (median 940.0 vs. 525.0 mg/day, *P* = 0.062). However, no statistically significant differences were observed between the two groups for any of the following variables: age, sex, disease duration, Hoehn-Yahr stage (on state), MMSE score, or total LEDD prior to initiation (all *P* > 0.05).

### 3.2 Changes in LEDD and LCC dosages over time

With the initiation of LDp/CDp infusion therapy, the regimen of oral anti-Parkinson's medications was revised. Table 2 shows the changes in LEDD and LCC before initiation, immediately after initiation, and at 1, 3, 6, and 12 months thereafter. The median LCC prior to initiation was 600.0 mg/day, but it decreased dramatically to 0.0 [0.0–150.0] mg/day immediately after initiation. Subsequently, the dosage was adjusted in response to fluctuations in symptoms, and at 12 months, the median LCC dosage was maintained at 150.0 [37.5–300.0] mg/day. A Friedman test revealed a statistically significant decrease in oral LCC dosage throughout the entire study period (*P* < 0.001), and post hoc analysis confirmed that a highly statistically significant reduction persisted at the 12-month mark compared to pre-initiation levels (*P* < 0.001). Total LEDD increased slightly from 1169.0 mg/day before initiation to 1251.2 [1034.0–1562.0] mg/day immediately thereafter and reached 1354.8 [1135.1–1792.4] mg/day at 12 months (*P* < 0.05).

**Table 2:** Changes in LEDD, LCC, and infusion rates over time.

Time point	Total LEDD (mg/day)	Oral LCC (mg/day)	Standard-setting infusion rate (mL/h)	Low-setting infusion rate (mL/h)
	Median [IQR] (min–max)	Median [IQR] (min–max)	Median [IQR] (min–max)	Median [IQR] (min–max)
Before initiation (N = 21)	1169.0 [900.0–1475.0] (800.0–2374.2)	600.0 [400.0–800.0] (300.0–1270.0)	–	–
Immediately after initiation (N = 21)	1251.2 [1034.0–1562.0] (612.0–2471.0)	0.0 [0.0–150.0] (0.0–300.0)	0.28 [0.25–0.34] (0.15–0.44)	0.19 [0.15–0.22] (0.00–0.34)
1 month (N = 21)	1273.5 [1120.7–1523.6] (770.8–2181.6)	100.0 [0.0–150.0] (0.0–400.0)	0.28 [0.25–0.32] (0.16–0.44)	0.20 [0.17–0.26] (0.00–0.34)
3 months (N = 16)	1296.8 [1157.7–1592.1] (693.6–2183.8)	150.0 [0.0–150.0] (0.0–375.0)	0.27 [0.21–0.36] (0.17–0.44)	0.20 [0.18–0.25] (0.15–0.37)
6 months (*N* = 15)	1388.6 [1116.7–1621.1] (600.0–2333.8)	150.0 [0.0–225.0] (0.0–600.0)	0.25 [0.22–0.35] (0.17–0.44)	0.20 [0.18–0.23] (0.15–0.37)
12 months (N = 15)	1354.8 [1135.1–1792.4] (693.6–2333.8)	150.0 [37.5–300.0] (0.0–550.0)	0.27 [0.22–0.35] (0.18–0.47)	0.20 [0.18–0.23] (0.15–0.37)

Abbreviations: IQR, interquartile range; LCC, levodopa-containing compound; LEDD, levodopa equivalent daily dose.

All values are shown as median [interquartile range] (minimum–maximum). N indicates the number of patients with available data at each time point (available case analysis). The Friedman test and post hoc Wilcoxon signed-rank tests with Bonferroni correction were performed on the 15 patients with complete data at all six time points: the oral LCC dose decreased significantly across the observation period (Friedman *p* < 0.001; 12 months vs. before initiation, p < 0.001), and the total LEDD increased significantly from before initiation to 12 months (*p* < 0.05). A low-setting infusion rate of 0.00 mL/h indicates that the infusion was suspended overnight at the patient's request rather than continued at a reduced nocturnal rate.

### 3.3 Changes in infusion rates

Under our institution's protocol, two continuous infusion rate programs were configured: a “standard setting” for routine use and a “low setting” used at night and during periods of increased physical activity. The infusion rate for the standard setting changed from a median of 0.28 mL/h immediately after initiation to 0.28 mL/h after 1 month, 0.27 mL/h after 3 months, 0.25 mL/h after 6 months, and 0.27 [0.22–0.35] mL/h after 12 months, thus remaining generally stable throughout the year. In contrast, the median infusion rate for the low setting changed from 0.19 mL/h immediately after initiation to 0.20 mL/h at 3 months and 0.20 [0.18–0.23] mL/h at 12 months. Notably, the IQR of the settings showed a tendency for them to widen at the 12-month mark.

### 3.4 Trends in the occurrence of adverse events by time period

As part of the safety evaluation, we conducted a detailed analysis of the total number of psychiatric symptoms and skin-related adverse events occurring over time. The total number of psychiatric events was concentrated immediately after initiation (7 events) and at 1 month (6 events) and then subsequently decreased (Table 3). Hallucinations and visual hallucinations were observed at a consistent rate in 2–3 patients throughout the entire period. However, while restlessness and agitation were observed in 3 patients immediately after initiation of treatment, this decreased to one patient at the 1-month mark following minor adjustments to the infusion rate. In contrast, depressive symptoms occurred in 2 patients during the early phase of treatment (within one month). Although treatment was discontinued in both patients by the third month, this small number (*n* = 2) limits their interpretation as predictive factors for treatment dropout.

**Table 3:** Distribution of psychiatric and skin-related adverse events at each assessment time point.

Event	Immediately after initiation	1 month	3 months	6 months	12 months
	(*N* = 21)	(N = 21)	(*N* = 16)	(*N* = 15)	(N = 15)
*Psychiatric*					
Hallucinations and visual hallucinations	2	2	2	3	2
Agitation/restlessness	3	1	0	1	1
Depression	1	2	0⁎	0⁎	0⁎
Dopamine dysregulation syndrome	1	1	0	0	0
Delusions (e.g., delusions of jealousy)	0	0	1	0	0

*Skin-related*					
Pain/erythema	0	6	2	2	1
Induration	0	2	5	6	6
Cellulitis/suppuration	0	1	0	2	2

Values are the numbers of patients with each event at each assessment time point. N indicates the number of patients with available data at each time point.

⁎ Two patients who discontinued treatment within 1 month because of worsening psychiatric symptoms, including depression, were no longer under observation at these time points.

In contrast to psychiatric symptoms, skin-related adverse events showed marked cumulative changes associated with long-term treatment (Table 3). One month after initiation, pain and erythema were the primary findings in 6 patients; however, induration—reflecting fibrosis of the subcutaneous tissue—increased and persisted, rising from 2 patients at 1 month to 5 patients at 3 months, 6 patients at 6 months, and 6 patients at 12 months. Cellulitis/suppuration was observed in 1 patient at 1 month, followed by 2 patients each at 6 and 12 months. Based on CTCAE v5.0, of the 6 patients who developed induration, 4 had Grade 1 and 2 had Grade 2; both Grade 1 and Grade 2 cases were managed by rotating the injection site and providing renewed guidance on skin care. Cellulitis and suppuration of Grade 2 were observed in 2 patients during the observation period. Antibiotics were administered, and neither patient required discontinuation of this therapy (no patients required temporary interruption). All instances of pain and erythema were of Grade 1.

## 4 Discussion

### 4.1 Clinical significance of the one-year continuation rate and reduction in burden of oral LCC

The 1-year continuation rate for LDp/CDp continuous subcutaneous infusion therapy in the study patients with advanced PD was 76.2% (16/21 patients), and the oral LCC dose remained low, decreasing from a median of 600.0 mg/day prior to initiation to 150.0 mg/day at 12 months. Contrastingly, total LEDD increased from 1169.0 mg/day before initiation to 1354.8 mg/day at 12 months, which may reflect optimization of the baseline dose following the transition to continuous subcutaneous infusion and infusion rate adjustments made in response to disease progression. Because standardized longitudinal data on motor symptoms and QOL were unavailable, treatment continuation and reduced oral LCC doses cannot establish symptomatic benefits.

A previous multicenter real-world clinical study examined MDS-UPDRS and PDQ-39 results and treatment discontinuation up to 6 months after initiation [12]. In contrast, the novelty of the present study lies in the fact that it presented the following as a set of real-world clinical data: 1) 12-month treatment persistence; 2) a temporal pattern in which psychiatric symptoms were concentrated in the early stages of initiation, but skin-related adverse events accumulated over the medium- to long-term phase; and 3) an implementation protocol combining a relatively low initial infusion rate, multidisciplinary collaboration, symptom diaries, and photographic documentation of infusion sites. Among patients who continued beyond the first month of treatment, no treatment discontinuation due to skin-related adverse events was observed up to 12 months. However, the small sample size limits direct comparisons of continuation rates with other studies.

Although conventional device therapies such as LCIG and DBS offer significant improvement in motor complications, surgical invasiveness and management complexity pose barriers to their adoption [7], [8]. LDp/CDp may be a useful alternative as a “third DAT” that requires no surgical procedures. In international Phase III trials, LDp/CDp was reported to increase on time and reduce off time without causing troublesome dyskinesia [9], [10].

### 4.2 Pre-initiation oral LCC dose in the discontinuation group

In 5 patients, psychiatric symptoms that emerged or worsened shortly after initiation were recorded as the reason for discontinuation. The median pre-initiation oral LCC dose was 940.0 mg/day in the discontinuation group and 525.0 mg/day in the continuation group; however, the difference was not statistically significant (*P* = 0.062). This comparison is an exploratory analysis based on the 5 discontinuation cases, and as it compares multiple background factors, the possibility that these findings are coincidental cannot be ruled out. Based on these results, it is not possible to infer predictive ability for treatment discontinuation, risk stratification, or clinical thresholds. Changes in the pattern of dopaminergic stimulation accompanying the transition from pulsatile stimulation with oral medications to continuous dopaminergic stimulation may contribute to these psychiatric symptoms [3], [14], [15], [16].

### 4.3 Biphasic nature of adverse events and transition to the management phase

This study also showed that the pattern of adverse event onset revealed starkly contrasting temporal characteristics (timelines) between psychiatric symptoms and skin-related adverse events. Psychiatric symptoms such as hallucinations, agitation, and excitement peaked immediately after initiation and within one month and constituted the primary risk of treatment discontinuation in the early phase (and the direct main cause of dropout). However, in the group that continued treatment beyond this initial phase, psychiatric symptoms improved following fine-tuning of the infusion rate and adjustments to concomitant oral medications.

In contrast, skin-related adverse events such as pain, erythema, induration, and cellulitis tended to increase cumulatively and persist over the long term [10], [11], [17]. In particular, subcutaneous induration increased after 3 months and remained at a high level even at 6 and 12 months. We speculate that this long-term accumulation of subcutaneous fibrosis and induration may have contributed to severe infections, such as cellulitis and suppuration, which increased after 6 months. Because LDp/CDp involves high-concentration local exposure to levodopa, it is prone to causing aseptic inflammation and subcutaneous nodules [18], [19], which is thought to form the pathophysiological basis for secondary bacterial infections [20].

In other words, although skin-related adverse events associated with this therapy are unlikely to be a direct cause of early treatment discontinuation, they can potentially manifest later as a risk of serious local infections during the long-term maintenance phase. When determining treatment strategies for PD, the importance of appropriately assessing and managing non-motor symptoms, such as psychiatric symptoms, has been emphasized [21], and the biphasic nature of these adverse events provides clear, time-specific indicators for management protocols aimed at long-term continuation. The initial phase (up to 1 month) is the “phase focused on managing psychiatric symptoms (to prevent dropout) through infusion rate adjustment [22], whereas the mid-to-late phase (3 months to 1 year) is the phase focused on managing skin-related adverse events (to prevent secondary infections) through thorough rotation of infusion sites and re-education on skin care. It is essential to adjust strategies according to the stage of treatment. Recent reports on real-world clinical protocols also recommend that proactive skin care and preventive interventions provided through multidisciplinary collaboration are extremely important for long-term continuation [11], [23], and the present study's findings regarding this two-phase nature strongly support this.

### 4.4 Importance of individualized infusion rate settings

The IQR of standard infusion rate settings during long-term therapy expanded from 0.25 to 0.34 mL/h immediately after initiation to 0.22–0.35 mL/h after 12 months. The significant widening of the IQR of the settings in both directions by the 12-month mark is an extremely interesting characteristic in real-world clinical practice. This may reflect the precise individualization (tailored adjustment) of dosing settings performed during outpatient management to accommodate disease progression over the long term, individual skin absorption status, or residual diurnal fluctuations (such as wearing-off and dyskinesia) [22], [23], [24]. Previous reports also recommend individualized infusion rate adjustments during long-term maintenance of LDp/CDp. [25], [26]

### 4.5 Limitations

This study is a retrospective analysis of 21 Japanese patients at a single tertiary care facility selected after multidisciplinary assessment of eligibility based on cognitive function, mental status, functional ability, and available support. Individual effects of initiation during hospitalization, dose adjustments, operational guidance, and outpatient support for patients and caregivers could not be evaluated. Without a control group, this study cannot determine whether a low initial infusion rate or multidisciplinary support improved treatment persistence. The results cannot be directly generalized to the entire advanced-stage PD population, other healthcare settings, or other ethnic groups. Ethnic differences in physical build, skin characteristics, or related adverse events were not evaluated.

Second, a major limitation is the lack of standardized, longitudinal assessments of motor symptoms and QOL. Treatment continuation and reductions in oral LCC dosage therefore cannot establish symptomatic benefits. Discontinuation of COMT and MAO-B inhibitors and adjustments to other anti-PD medications coincided with the switch to LDp/CDp. Their effects on psychiatric symptoms and clinical course could not be isolated from those of LDp/CDp, and the impact of concomitant medication withdrawal on motor symptoms could not be quantified.

Third, adverse events were identified only through retrospective medical record reviews and thus may have been under-recorded and underestimated. Later assessments excluded patients who discontinued treatment, potentially introducing survivor bias into comparisons of adverse events across time. More frequent observation during initial hospitalization than during outpatient care may have introduced ascertainment bias and the apparent concentration of psychiatric symptoms early in treatment. Photographs of all puncture sites supported assessment of skin-related events, but differences in detection or documentation across time cannot be excluded. Incomplete medical records may also have affected accuracy of severity assessment.

Finally, baseline between-group comparisons were exploratory, and statistical power was limited. Although risk factors or predictors of treatment discontinuation were not established, non-significant differences do not exclude clinically meaningful associations. Future multicenter, prospective studies incorporating standardized efficacy and safety assessments are needed to clarify factors related to 12-month treatment persistence and early dropout and temporal patterns of adverse events.

## 5 Conclusion

Based on the real-world clinical data from this study, continuous subcutaneous LDp/CDp infusion therapy for advanced PD showed favorable treatment persistence and a sustained reduction in oral LCC burden over 12 months. Furthermore, the data revealed temporal characteristics in which psychiatric symptoms were concentrated in the early phase of initiation, whereas skin-related adverse events accumulated over the medium- to long-term phase. All treatment discontinuations were directly attributable to psychiatric symptoms that emerged or worsened within 1 month of initiation. In our facility's experience, the focus of care shifted depending on the stage of treatment. Specifically, in the early stages, the emphasis was on monitoring psychiatric symptoms, whereas in the medium- to long-term stages, the focus shifted to preventing skin-related events and infections.

## CRediT authorship contribution statement

**Naoki Takao:** Writing – original draft, Visualization, Validation, Methodology, Investigation, Conceptualization. **Makoto Shiraishi:** Writing – original draft, Visualization, Validation, Supervision, Project administration, Methodology, Investigation, Conceptualization. **Hidetaka Naganuma:** Writing – review & editing. **Takayuki Kawazawa:** Writing – review & editing. **Sakae Hino:** Writing – review & editing. **Yoshihisa Yamano:** Writing – original draft, Visualization, Validation, Supervision, Project administration, Methodology, Investigation, Conceptualization.

## Declaration of competing interest

The authors declare the following financial interests/personal relationships which may be considered as potential competing interests: [Naoki Takao has received speaker honoraria from AbbVie GK and Eisai Co., Ltd. Makoto Shiraishi has received speaker honoraria from AbbVie GK, Kyowa Kirin Co., Ltd., Takeda Pharmaceutical Company Limited, Ono Pharmaceutical Co., Ltd., Eisai Co., Ltd., and Insightec Ltd. Yoshihisa Yamano has received speaker honoraria from LSI Medience Corporation, NTT Precision Medicine Corporation, Human Connect Co., Ltd., and Kaishin Co., Ltd. Hidetaka Naganuma, Takayuki Kawazawa, and Sakae Hino have no additional disclosures to report].
